# Preparation and Characterization of Graphite–SiO_2_ Composites for Thermal Storage Cement-Based Materials

**DOI:** 10.3390/ma17122880

**Published:** 2024-06-12

**Authors:** Chenhao He, Xiangguo Li, Yang Lv, Jianming Dan, Haitian Yan, Xiangqin Shi

**Affiliations:** 1 State Key Laboratory of Silicate Materials for Architectures, Wuhan University of Technology, Wuhan 430070, China; hch@whut.edu.cn (C.H.); yang.lv@whut.edu.cn (Y.L.); yanhaitian0907@163.com (H.Y.); sxq18229361754@163.com (X.S.); 2 State Key Laboratory Incubation Base for Green Processing of Chemical Engineering, School of Chemistry and Chemical Engineering, Shihezi University, Shihezi 832003, China; danjmxj@163.com

**Keywords:** core-shell structure, thermal storage cement-based materials, thermal conductivity, carbon material

## Abstract

Thermal storage cement-based materials, formed by integrating phase change materials into cementitious materials, exhibit significant potential as energy storage materials. However, poor thermal conductivity severely limits the development and application of these materials. In this study, an amorphous SiO_2_ shell is encapsulated on a graphite surface to create a novel thermally modified admixture (C@SiO_2_). This material exhibits excellent thermal conductivity, and the surface-encapsulated amorphous SiO_2_ enhances its bond with cement. Further, C@SiO_2_ was added to the thermal storage cement-based materials at different volume ratios. The effects of C@SiO_2_ were evaluated by measuring the fluidity, thermal conductivity, phase change properties, temperature change, and compressive strength of various thermal storage cement-based materials. The results indicate that the newly designed thermal storage cement-based material with 10 vol% C@SiO_2_ increases the thermal conductivity coefficient by 63.6% and the latent heat of phase transition by 11.2% compared to common thermal storage cement-based materials. Moreover, C@SiO_2_ does not significantly impact the fluidity and compressive strength of the thermal storage cement-based material. This study suggests that C@SiO_2_ is a promising additive for enhancing thermal conductivity in thermal storage cement-based materials. The newly designed thermal storage cement-based material with 10 vol% C@SiO_2_ is a promising candidate for energy storage applications.

## 1. Introduction

With the reduction in fossil fuel use and the environmental problems caused by energy consumption, energy storage is receiving more and more attention around the world as a means to achieve efficient energy utilization [1]. Among the various energy storage methods, thermal energy storage is the most effective method to regulate energy supply. Currently, energy storage materials have shown potential applications in the fields of building energy efficiency, industrial waste heat recovery, and solar energy utilization [1,2,3,4]. Common thermal energy storage methods can be categorized into sensible heat storage, latent heat storage, and thermochemical storage. Latent heat storage based on phase change materials (PCMs) has attracted much research attention due to its large energy storage capacity and minimal temperature change during heat storage and exothermic processes [4,5,6,7]. PCMs are widely used, and their application in construction materials is a prominent example [8,9,10]. Studies have shown that thermal storage cementitious materials (TSCMs) formed by encapsulating PCMs in cementitious materials are characterized by high heat capacity, low hazard, and easy accessibility [11,12,13]. In addition, TSCM can effectively mitigate daily temperature variations, reduce energy demand, and absorb and release thermal energy at an almost constant temperature, making it an excellent energy storage material [14,15,16,17,18,19].

To prepare TSCMs, several methods have been proposed by researchers around the world. The first method is to directly mix PCMs with cementitious materials [20,21]. While the PCMs can remain solid during the preparation process of the TSCMs by this method, they transition into a liquid state once the ambient temperature exceeds their phase change temperature. This leads to the PCMs leaking through the pores in the TSCMs, ultimately causing the loss of their heat storage capability. The second method is to absorb the PCMs by capillary force by immersing the molded cementitious material components into the liquid phase PCMs. However, this method may affect the durability of the cementitious material and hence its practicality [22,23]. The third method is to first encapsulate the PCMs using a stable shell, thus obtaining phase change microcapsules (MPCMs) with a core-shell structure. The MPCM is then added to the gelling material to prepare TSCM [16,24,25,26]. This TSCM has good durability, prevents PCM leakage, and ensures the thermal storage capacity of the TSCM [17,27,28]. Therefore, the preparation of TSCMs using phase change microcapsules is considered to be the most promising method.

Compared with ordinary PCMs, MPCMs generally have good mechanical properties and durability. And the thicker the shell of the MPCM, the better these properties are [29,30,31]. However, the presence of the shell can adversely affect the thermal conductivity of the MPCM [32,33,34]. As a result, the thermal conductivity of TSCMs is much lower than that of ordinary cement [35,36,37]. The problem of poor thermal conductivity seriously affects the heat storage and heat release rate of TSCMs, thus limiting their wide application as an energy storage material [38,39]. To address this issue, researchers have explored the use of various additives in TSCMs to improve their thermal conductivity [40,41]. The results have shown that carbon-based materials (e.g., carbon nanotubes, expanded graphite, and graphene) can effectively improve the thermal conductivity of TSCMs [6,42,43,44,45,46]. However, these materials are expensive and their extensive use in TSCMs may lead to a significant increase in the preparation cost, thus limiting their application.

Graphite is a widely used carbon material that is inexpensive and has good thermal conductivity. Studies have shown that incorporating graphite into cement can effectively improve the thermal conductivity of the cement paste [47,48]. However, graphite is an inert material and does not actively participate in cement hydration. Moreover, graphite has poor hydrophilicity and is prone to producing pores in the cement paste. Therefore, graphite has a serious negative impact on the mechanical properties and workability of cement pastes [49]. Currently, the main method of utilizing graphite in the construction industry is to convert it into materials such as graphene oxide, which is then added to the cement paste [50]. However, the preparation process of graphene oxide is costly, and a large number of hazardous and corrosive chemicals are used. All these reasons limit the wide application of graphite in construction materials [51,52].

In this study, we made a new thermally modified admixture (C@SiO_2_) using graphite as the raw material. This admixture has a core-shell structure, with an amorphous SiO_2_ shell and a graphite core. The prepared C@SiO_2_ has similar thermal conductivity to graphite and can solve the problem of poor bonding between graphite and cement. In addition, C@SiO_2_ is used in a TSCM to form a new TSCM with good thermal conductivity. The physical phase composition, microstructure, and thermal conductivity of C@SiO_2_ were well evaluated. For the new TSCM with good thermal conductivity, the workability, mechanical properties, and thermal performance were examined, which is helpful to move forward the practical application of TSCMs in energy storage materials.

## 2. Materials and Experiment

### 2.1. Materials

The graphite used in this experiment was supplied by China Minmetals Corporation (Beijing, China), and had an average particle size of 1.76 μm. The phase change material used in the experiments was n-octadecane (analytical grade), purchased from Shanghai Aladdin Biochemical Technology Co., Ltd. (Shanghai, China). Tetraethyl orthosilicate (TEOS), cetyltrimethylammonium bromide (CTAB), anhydrous ethanol, hydrochloric acid, and ammonium hydroxide are of analytical grade and were purchased from Shanghai Aladdin Biochemical Technology Co., Ltd.

**Figure 1:**
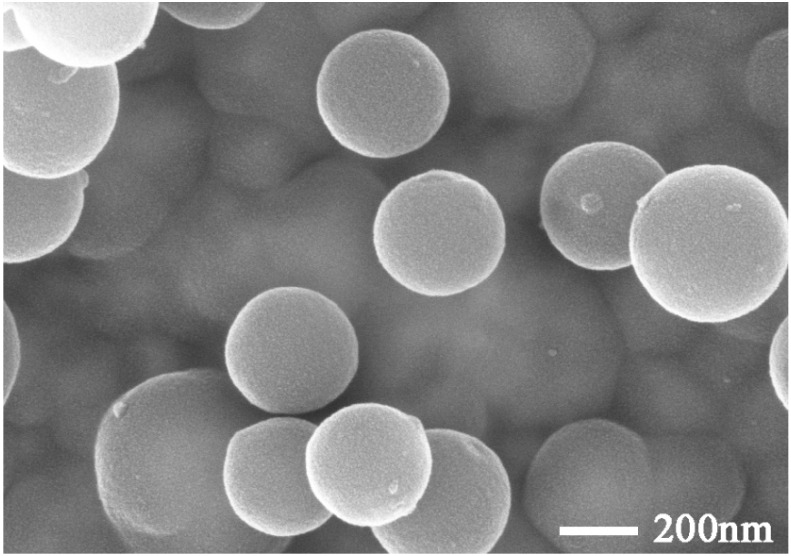
Microscopic image of MPCMs.

Based on previous studies [53,54,55], 4 g of n-octadecane, 6 g of TEOS, 180 g of distilled water, 100 g of anhydrous ethanol, and 0.82 g of CTAB were added to a three-necked flask. The mixture was then emulsified using a dispersator for 30 min at 800 rpm to form an emulsion. Subsequently, the emulsion was transferred to an ultrasonic crusher with an ultrasonic power of 750 W for 10 min to form a stable microemulsion. A total of 2 mL of ammonia was added to the emulsion, followed by continuous reaction for 24 h. The reaction mixture was then filtered and washed three times with distilled water. The obtained microcapsules were named MPCMs. The prepared MPCMs presented a spherical shape, smooth surface, and good dispersity (as shown in Figure 1). The individual properties are shown in Table 1.

The cement used was a typical commercial Ordinary Portland cement (P⋅O42.5), supplied by Huaxin Cement Technology Management (Wuhan, China) Co., Ltd. That complies with the requirements of specification GB175-2007 [56]. Its chemical composition is shown in Table 2.

### 2.2. Experiments

#### 2.2.1. Preparation of MPCMs and C@SiO_2_

In accordance with the Stöber method [57], a certain amount of graphite, 180 g of distilled water, and 100 g of anhydrous ethanol were added to a three-necked flask. The mixture was then emulsified using a dispersator for 30 min at 1200 rpm. The solution pH was adjusted to 11 with ammonia, then TEOS was added slowly dropwise, followed by continuous reaction for 6 h. The reaction mixture was then filtered and washed three times with distilled water. The obtained mixture was named C@SiO_2_. The specific amounts of each material are shown in Table 3.

#### 2.2.2. Preparation of Thermal Storage Cement Mortars

Cement, MPCMs, and C@SiO_2_ were premixed for 1 min in an agitator. Next, river sand and water were added to the above mixture and mixed for 2 min to produce TSCMs. The mortars were mixed with a sand-to-binder weight ratio of 3 and a water-to-binder ratio of 0.5 according to GB/T17671–2021, test method of cement mortar strength (ISO method) (equivalent to ISO 679-2009) [58]. To ensure that the MPCMs do not adversely affect the strength of the TSCMs, the MPCM substitution rate was determined to be 10% by volume of cement [54]. Four C@SiO_2_ replacement ratios (5%, 10%, 15%, and 20% by volume of cement) were selected. The mix proportion of cement mortars is depicted in Table 4.

### 2.3. Characterization Methods

#### 2.3.1. Microscopic Morphology

The microstructure of samples was observed using a JSM-IT300 Schottky field emission scanning electron microscope (SEM, Jeol, Tokyo, Japan) at an extra high tension (EHT) of 15–20 kV and a working distance (WD) of 10 mm. The elemental composition of the samples was also analyzed using its additional Ultim Max 65 Energy Dispersive Spectrometer (EDS, Oxford Instruments, Oxford, UK).

#### 2.3.2. Phase Change Properties

The phase change properties of samples were determined in accordance with GB/T 13464-2008, thermal analysis test methods for the thermal stability of materials (equivalence with ASTM E2550) [59]. Equipment used for testing was a DSC2500 differential scanning (DSC, TA, New Castle, DE, USA). The sample weight was about 5 mg, in a nitrogen atmosphere, the test temperature range was −10 °C to 50 °C to −10 °C, and the heating rate was 50 °C/min.

#### 2.3.3. Workability

After the mixing of the TSCMs, the workability was evaluated according to GB/T2419-2005, test method for fluidity of cement mortar (equivalent to ASTM C1437) [60]. The mixed mortar was transferred to a cone mold by two layers. The mixture was vibrated once per second. The maximum diameter and vertical diameter of the sample were measured with a caliper, and the average value was calculated. Each sample was tested three times.

#### 2.3.4. Mechanical Property Test

To measure the compressive strength of cement mortars, the mixtures were cast into 40 mm × 40 mm × 40 mm molds in accordance with GB/T17671–2021, test method of cement mortar strength (ISO method) (equivalent to ISO 679-2009) [58]. The specimens were cured in standard conditions with a temperature of 20 ± 1 °C and a relative humidity of 95%. The compressive strength test was performed using a TYE-300F Flexural and Compressive Testing Machine (JIANYI, Wuxi, China) for Cementitious Sand with a loading rate of 2.4 kN/s.

#### 2.3.5. Thermal Conductivity

The thermal conductivity of samples was determined in accordance with GB/T 13464-2008, thermal analysis test methods for thermal stability of materials (equivalent to ASTM E2550) [59]. Equipment used for testing was a TPS 2500S Thermal conductivity analyzer (Hot Disk, Gothenburg, Sweden).

Additionally, an LC-DB-XDA thermostatic heating table (LICHEN, Shanghai, China) and an infrared camera (HIKMICRO, Hangzhou, China) were used to study the temperature changes in the TSCMs during the warming process. Before the test started, the molded and dried TSCM boards were first placed in a constant temperature and humidity chamber (set at 20 °C and 0 humidity) for one day to ensure that the temperature of the TSCM boards was 20 °C at the start of the test. During the test, the TSCM boards were placed on a thermostatically heated bench at 50 °C, and a polystyrene foam sheet was used to cover the sides and the top of the TSCM boards to prevent heat loss. The temperature of the TSCM boards was then measured using an infrared thermography camera every minute until its temperature increased to 50 °C.

## 3. Results and Discussion

### 3.1. Preparation and Properties of C@SiO_2_

**Figure 2:**
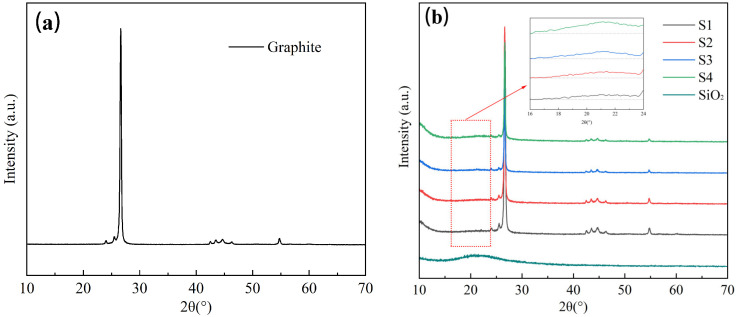
XRD test results of graphite (**a**) and C@SiO_2_ (**b**).

C@SiO_2_ was prepared according to the method described in Section 2.3, and its chemical composition was analyzed using an X-ray diffractometer, with the results shown in Figure 2.

As shown in Figure 2, the XRD images of S1, S2, S3, and S4 clearly exhibit distinguishable diffraction peaks at 2θ = 26.61°, 43.02°, 43.45°, 44.67°, 46.32°, and 54.81°, which align with the positions observed in the XRD images of graphite. However, no sharp diffraction peaks are observed at other positions, indicating that graphite is the primary constituent of C@SiO_2_. Additionally, the XRD images of S1, S2, S3, and S4 show a weak broad hump in the range of 2θ = 15°~30°, corresponding to the diffraction pattern of amorphous SiO_2_. This suggests the presence of SiO_2_ generated by the TEOS reaction in the samples. Furthermore, the peaks of each sample exhibit the same position in the diffraction pattern, suggesting a physical bonding mode between SiO_2_ and graphite without chemical changes. However, accurately determining the variation in SiO_2_ content in different samples is challenging because XRD cannot intuitively measure the bonding mode of SiO_2_ with graphite, and amorphous SiO_2_ lacks a crystal structure with obvious sharp diffraction peaks. Therefore, the microscopic morphologies of the graphite and C@SiO_2_ samples were analyzed by SEM and the elemental composition of C@SiO_2_ was analyzed by EDS.

**Figure 3:**
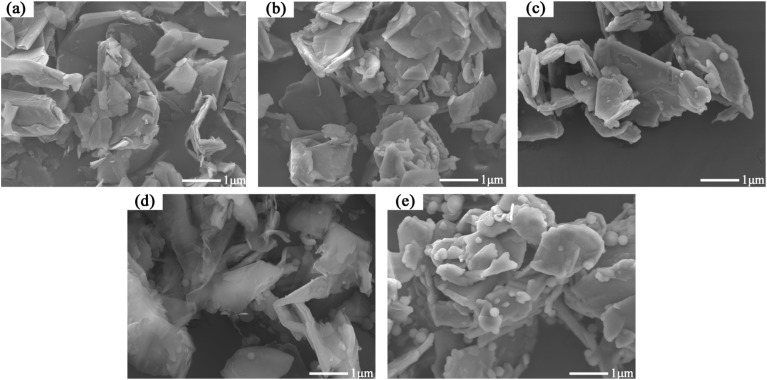
SEM test results of C@SiO_2_ ((**a**): Graphite, (**b**): S1, (**c**): S2, (**d**): S3, (**e**): S4).

The SEM test results are shown in Figure 3. Compared to Figure 3a, the boundaries of the samples in Figure 3b–e are smoother. Furthermore, the boundaries of the samples become smoother with increasing TEOS usage. In addition, a large number of smooth spheres can be observed in the images of S3 and S4, while such phenomena are almost absent in the images of S1 and S2.

**Figure 4:**
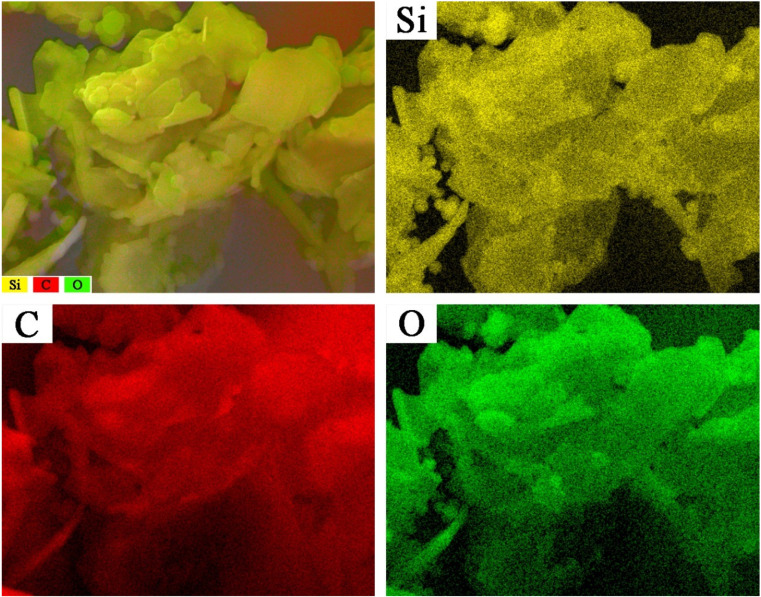
EDS test results for C@SiO_2._

To elucidate the reasons for these phenomena, the elemental composition of S4 was analyzed using SEM-EDS as an example. The results are presented in Figure 4 and Table 5.

According to the EDS test results in Figure 4, the C@SiO_2_ samples are mainly composed of three elements: C, Si, and O. The contents of Si and O elements in the samples gradually increase with an increase in TEOS doping in the raw materials. In addition, smooth spheres can be clearly observed in the Si elemental distribution map, and this phenomenon is also evident at the same location in the O elemental distribution map. However, it is difficult to see smooth spheres in the C element distribution. Therefore, it can be assumed that both the material wrapped around the graphite surface and the smooth spheres in Figure 3 are composed of SiO_2_, and the SiO_2_ content in C@SiO_2_ increases gradually with an increase in TEOS doping in the raw material. When the mass of TEOS used in the preparation of C@SiO_2_ exceeds the mass of graphite, there is an excess of SiO_2_ in C@SiO_2_. The SiO_2_ cannot be fully encapsulated on the graphite surface and the excess exists in the form of nano-SiO_2_ spheres.

**Figure 5:**
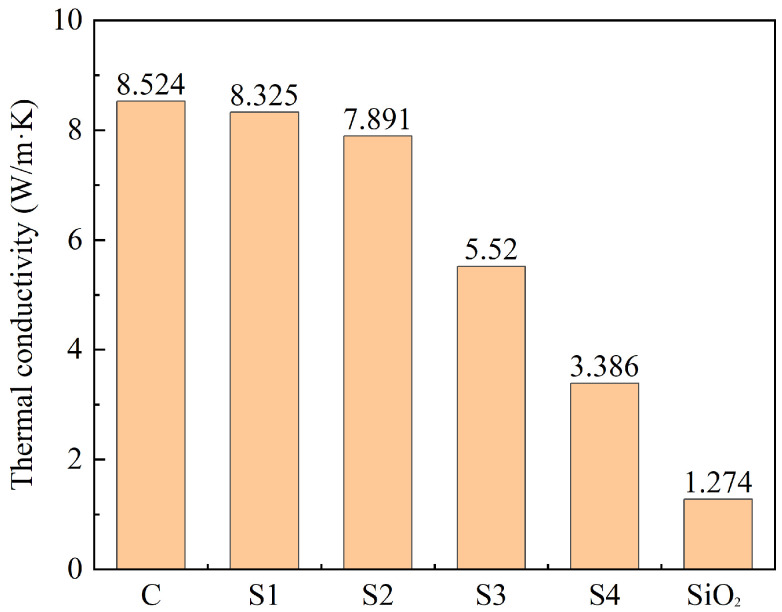
Thermal conductivity coefficients of graphite (C), SiO_2_, and C@SiO_2._

To determine the optimal ratio of graphite to TEOS, the thermal conductivity coefficients of graphite, C@SiO_2_, and nano-SiO_2_ prepared by the Stöber method were tested, with the results shown in Figure 5.

According to Figure 5, the thermal conductivity coefficient of C@SiO_2_ is lower than that of graphite (C), and the larger the amount of TEOS, the smaller the thermal conductivity coefficient of C@SiO_2_. The thermal conductivity coefficients of S1 and S2 are 97.67% and 92.58% of that of graphite, respectively. This indicates that when the amount of TEOS in the raw material for C@SiO_2_ is less than that of graphite, the thermal conductivity coefficient of C@SiO_2_ is not much different from that of graphite. Conversely, the thermal conductivities of S3 and S4 are 64.76% and 39.72% of that of graphite, respectively, indicating a significant deterioration in the thermal conductivity coefficient when the amount of TEOS in the raw material for the preparation of C@SiO_2_ is too high.

Compared to graphite, SiO_2_ has a very low thermal conductivity coefficient of 1.274 W/m-K. When SiO_2_ is encapsulated on the graphite surface, it hinders heat transfer between graphite particles. Therefore, the thermal conductivity coefficient of C@SiO_2_ is lower than that of graphite. However, when the thickness of SiO_2_ encapsulated on the graphite surface is thin, the effect on the thermal conductivity coefficient is less pronounced. As a result, the thermal conductivity coefficient of S1 is similar to that of graphite, while the thermal conductivity coefficient of S2 is only 7.42% lower compared to graphite. Combined with Figure 3 and Figure 4, these results show that a substantial amount of SiO_2_ is present on the surface of the S3 and S4 samples, and numerous SiO_2_ spheres are observed. This leads to a significant decrease in the thermal conductivity coefficients of S3 and S4. According to Table 1, the thermal conductivity coefficient of MPCM is only 0.48 W/m-K, which is much lower than that of C@SiO_2_. Therefore, although the thermal conductivity coefficient of C@SiO_2_ is lower than that of graphite, it can still be used to improve the thermal conductivity of TSCM.

The combined SEM-EDS and thermal conductivity test results show that S2 is the best C@SiO_2_ in terms of overall performance. It has excellent thermal conductivity along with a good core-shell structure, where the core material is graphite and the shell material is amorphous SiO_2_. Therefore, combined with the results shown in Table 3, the optimum mass ratio of graphite to TEOS for the preparation of C@SiO_2_ is 1:1.

### 3.2. Effect of C@SiO_2_ on Workability of TSCM

**Figure 6:**
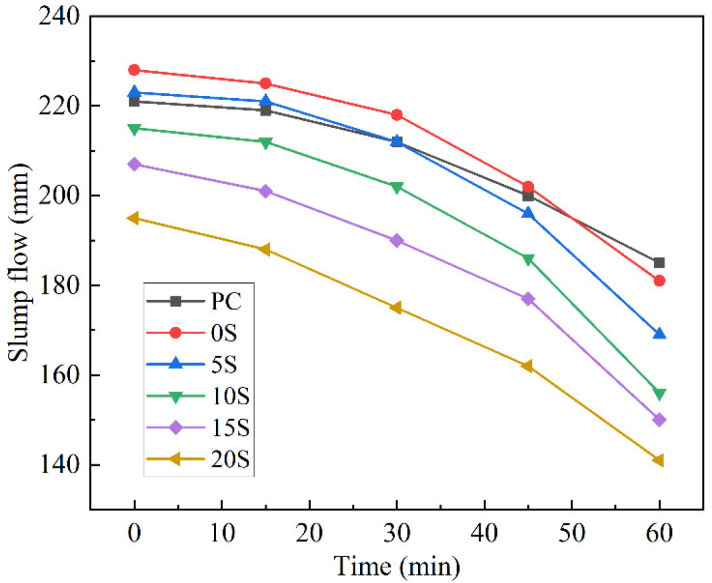
Fluidity test results of ordinary cement mortar and TSCMs.

To elucidate the effect of C@SiO_2_ content on the workability of TSCMs, TSCMs were prepared using the method described in Section 2.2.2. TSCMs were prepared using S2 as an example based on the analytical results in Section 3.1. The effect of C@SiO_2_ dosage on the fluidity of cement mortar was evaluated, with the results presented in Figure 6. The initial fluidity of 0S was 228 mm, which was higher than that of PC at 221 mm. This is attributed to the fact that MPCMs can improve the fluidity of cement mortar [54]. However, the initial fluidity of the TSCMs decreases with an increase in C@SiO_2_ doping. The higher the C@SiO_2_ dosage, the more obvious the decrease in initial fluidity. Specifically, the initial fluidity of the TSCMs decreased to 223 mm, 216 mm, 207 mm, and 195 mm at C@SiO_2_ doping levels of 5%, 10%, 15%, and 20%, respectively. This indicates that C@SiO_2_ does not exert the same effect as MPCMs in cement paste. In general, the fluidity of cementitious materials is greatly influenced by the additives. The smaller the density and fineness, and the larger the specific surface area of the admixtures, the more prominent the adsorption of free water in the cement paste. As a result, such admixtures lead to a decrease in the fluidity of the cement paste. Both MPCMs and C@SiO_2_ cause this phenomenon in cement paste. Additionally, the morphology of admixtures significantly impacts the fluidity of cement paste. Spherical admixtures have a lubricating effect on the cement paste and improve its flowability. As shown in Figure 1 and Figure 3, MPCMs have a regular spherical shape while C@SiO_2_ has an irregular shape. MPCMs improve the fluidity of TSCMs while C@SiO_2_ reduces the fluidity of TSCMs.

From the perspective of flow loss, the initial fluidity of PC was lower than that of 0S and 5S. However, the 60 min fluidity of PC was higher than that of all five TSCMs. In other words, over time, the fluidity of the TSCMs decreased at a faster rate than that of ordinary cement pastes. As both C@SiO_2_ and MPCMs contain amorphous SiO_2_ on their surfaces, they can accelerate the hydration process of cement in cement pastes. Consequently, the fluidity loss of the TSCMs was significantly higher than that of ordinary cement mortar over time. Considering the above analyses, the dosage of C@SiO_2_ in TSCMs should not be excessively high, as it may deteriorate the working performance of the TSCMs.

### 3.3. Effect of C@SiO_2_ on Thermal Storage Performance of TSCM

**Figure 7:**
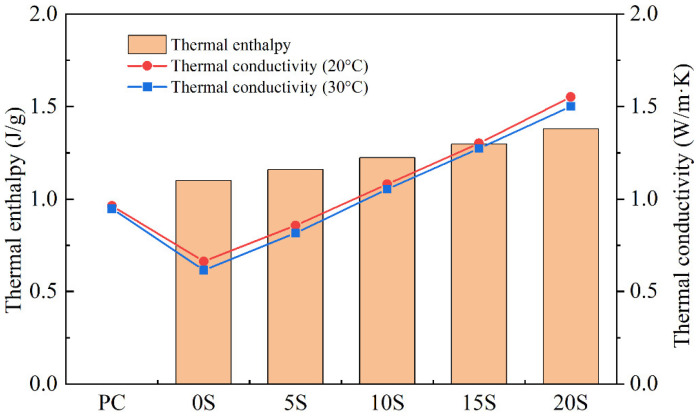
Phase transition latent heat and thermal conductivity coefficients at 20 °C and 30 °C of ordinary cement mortar and TSCMs.

The phase transition latent heat and the thermal conductivity coefficients at 20 °C and 30 °C of different samples were determined according to Section 2.3.5, as depicted in Figure 7. The test results reveal that the phase transition latent heat of the TSCMs slightly increases with an increase in C@SiO_2_ doping. Since C@SiO_2_ is not a phase change material, its addition to TSCMs does not increase the total thermal storage capacity of the TSCMs. However, the density of C@SiO_2_ is about 0.8 g/cm^3^, while the density of cement paste is about 2.2 g/cm^3^. When C@SiO_2_ replaces the same volume of cement in a TSCM, this results in a lower mass of the TSCM. Consequently, the latent heat of phase change per unit mass of TSCM increases with the same amount of MPCMs. Hence, it can be inferred that C@SiO_2_ can enhance the heat storage capacity per unit mass of TSCM.

In contrast to the phase transition latent heat, the influence of C@SiO_2_ on the thermal conductivities of the TSCMs is substantial. For instance, the thermal conductivity coefficient at 20 °C is 0.66 W/m·K for TSCM without C@SiO_2_, which increases to 0.86 W/m·K, 1.08 W/m·K, 1.30 W/m·K, and 1.55 W/m·K for TSCMs with C@SiO_2_ doping of 5%, 10%, 15%, and 20%, respectively. This pattern of variation also holds true for the thermal conductivity coefficients at 30 °C. Thus, C@SiO_2_ can significantly enhance the thermal conductivity coefficients of TSCMs irrespective of whether the temperature is above or below the phase transition temperature of the TSCM. When the dosage of C@SiO_2_ is 5%, the thermal conductivity coefficient of the TSCM is comparable to that of PC, and it surpasses that of PC as the dosage of C@SiO_2_ continues to increase. Therefore, C@SiO_2_ effectively addresses the issue of poor thermal conductivity of cementitious materials caused by MPCMs.

**Figure 8:**
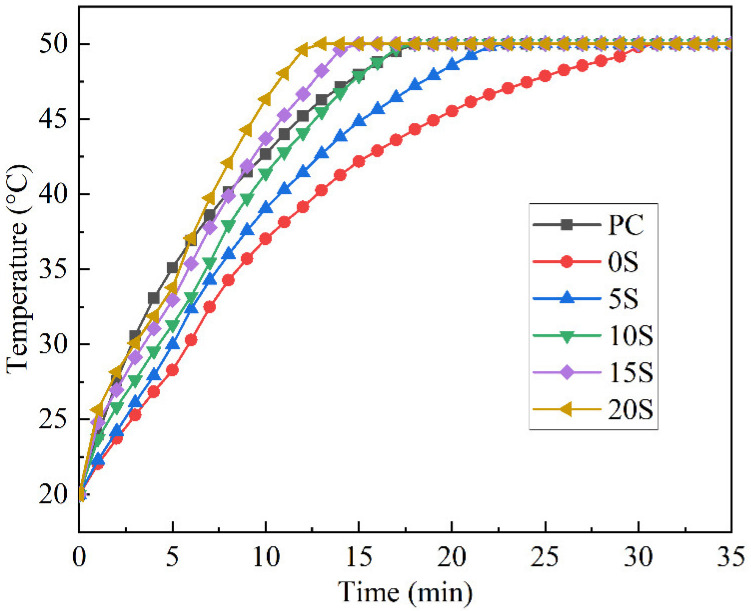
Temperature variation of ordinary cement mortar and TSCMs during the heating process.

To intuitively assess the effect of C@SiO_2_ on the thermal performance of TSCM storage, the thermal performance of the TSCM plates during the warming process was evaluated using the method described in Section 2.3.2 and Section 2.3.5. The temperature change curves of different samples are illustrated in Figure 8.

It is evident that when the C@SiO_2_ doping of TSCM plates is below 10%, the time required for the TSCM to warm up to 50 °C exceeds that of PC. Conversely, when the C@SiO_2_ doping exceeds 10%, the time required for the TSCM to warm up to 50 °C is less than that of PC. This suggests that C@SiO_2_ can expedite the warming rate of TSCMs, with the enhancement in temperature increase rate becoming more pronounced as the degree of C@SiO_2_ doping increases. Since there is a definite phase transition temperature range for MPCMs, the rapid temperature increase enables the TSCM to reach the phase transition temperature of the MPCMs sooner, thus facilitating the phase transition heat storage process. Therefore, it can be inferred that C@SiO_2_ enhances the thermal storage efficiency of TSCMs.

It should be noted that the thermal conductivity coefficient of a TSCM is not entirely correlated with its rate of temperature increase. As shown in Figure 7, the thermal conductivity coefficient of 10S is higher than that of PC, but the time required for the temperatures of both materials to rise to 50 °C is almost the same, with the temperature of PC being higher than that of 10S for most of the time during the warming process. This indicates a difference between the warming process of TSCMs and that of ordinary cement mortar. This disparity is also evident in the warming processes of 15S and 20S. Initially, the temperatures of 15S and 20S are higher than that of PC at 1 min, indicating a faster heating rate compared to PC, after which the heating rate declines notably. By 3 min, the temperature of PC surpasses that of 15S and 20S, a trend that persists until 6 min. At 7 min, the temperature of 20S exceeds that of PC, and by 9 min, the temperature of 15S surpasses that of PC. Subsequently, the temperature and heating rate of both 15S and 20S are higher than that of PC, attributable to the heat absorption by the MPCMs in the TSCMs.

At the onset of the heating process, the temperature of the TSCMs is 20 °C, which does not reach the phase transition temperature range of MPCMs, and thus is insufficient to stimulate the phase transition. After heating for 1 min, the temperatures of 15S and 20S exceed the starting melting point of MPCMs, initiating the heat storage through the phase transition process, resulting in a lower heating rate for 15S and 20S. With the prolongation of heating time, most of the MPCMs in the TSCMs melt into a liquid state, and the heating rate of the TSCMs gradually increases. This phenomenon is evident in 10S, 15S, and 20S, which have higher thermal conductivity. In contrast, 0S and 5S, with lower thermal conductivity, do not exhibit significant changes in heating rate due to their inherently lower heating rate and longer duration of the heat storage process. Therefore, no discernible rule of change similar to that of 10S, 15S, and 20S is observed in the warming process of 0S and 5S.

In conclusion, C@SiO_2_ can enhance the heat storage capacity per unit mass of TSCM by decreasing the TSCM’s density and improve its heat storage efficiency by increasing the thermal conductivity of the TSCM. Consequently, C@SiO_2_ exhibits a favorable effect on the thermal storage capacity of TSCMs, with higher C@SiO_2_ doping resulting in the TSCMs having a stronger thermal storage capacity.

### 3.4. Effect of C@SiO_2_ on Compressive Strength of TSCMs

**Figure 9:**
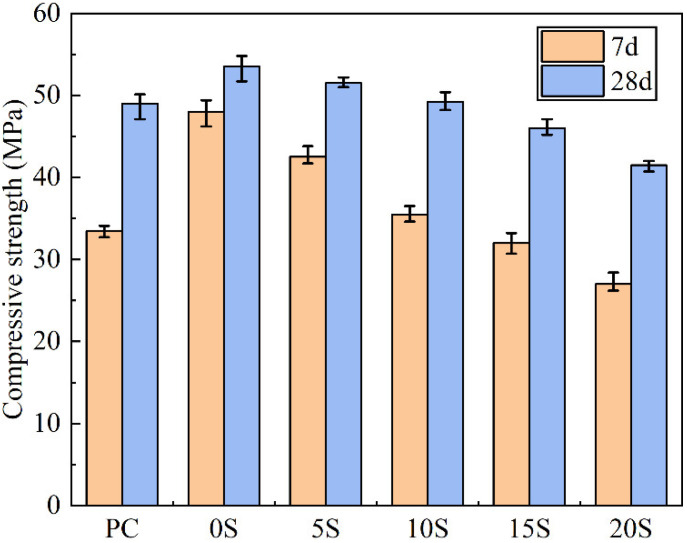
Compressive strength of ordinary cement mortar and TSCMs.

To comprehend the effect of C@SiO_2_ on the mechanical properties of TSCMs, the compressive strengths of TSCMs containing varying percentages of C@SiO_2_ were evaluated. As depicted in Figure 9, the 7-day and 28-day compressive strengths of TSCMs without C@SiO_2_ exceeded those of ordinary cement mortar. This superiority is primarily attributed to the ability of MPCMs to exert a microaggregate effect in the cement paste. Furthermore, since the surface of the MPCMs comprises amorphous SiO_2_, this can augment the nucleation point of hydration product precipitation, thereby enhancing the compressive strength, particularly the early strength, of the cement paste [54]. According to the analyses in Section 3.1, the surface of C@SiO_2_ is mainly composed of amorphous SiO_2_, similar to the shell component of the MPCMs. Therefore, C@SiO_2_ is also able to promote hydration and increase the compressive strength of the cement paste.

However, as indicated in Figure 9, the compressive strength of TSCMs containing C@SiO_2_ is markedly lower than that of TSCMs without C@SiO_2_, and the compressive strength of TSCMs declines progressively with the increasing proportion of C@SiO_2_ utilized. This phenomenon can be attributed to two primary reasons. Firstly, unlike MPCMs, which comprise smooth spherical particles approximately 200 nm in size, C@SiO_2_ exhibits an irregular shape with an average particle size of 1.76 μm, rendering it challenging for C@SiO_2_ to exert a robust microaggregate effect in the cement paste akin to MPCMs. Secondly, since 10% of the cement has already been replaced by MPCMs in the TSCMs, the utilization of C@SiO_2_ to further replace cement results in a reduction in the amount of hydration products in the slurry. With increasing C@SiO_2_ dosage, the total amount of cement in the slurry decreases, leading to a gradual reduction in the quantity of hydration products. Consequently, the compressive strength of TSCMs with higher C@SiO_2_ dosage experiences a significant decrease.

Therefore, it can be concluded that the effect of C@SiO_2_ on the strength of cement paste has both positive and negative aspects, with its negative impact on the strength of TSCMs becoming more pronounced with increasing C@SiO_2_ doping. Considering Figure 9, the strengths of the TSCMs surpass that of PC when the C@SiO_2_ doping is below 10%. Hence, from a mechanical properties perspective, C@SiO_2_ doping should not exceed 10%, as higher levels may adversely affect the compressive strength of the cement paste.

Based on the analysis of workability, thermal, and mechanical properties, the optimal dosage of C@SiO_2_ is determined to be 10%.

## 4. Conclusions

In this study, a novel TSCM with favorable thermal and mechanical properties was developed by incorporating SiO_2_-encapsulated graphite, effectively addressing the poor thermal conductivity of TSCMs. The following conclusions can be drawn:

A graphite–SiO_2_ composite (C@SiO_2_) with a core-shell structure was successfully prepared using graphite and TEOS. The core of C@SiO_2_ is graphite and the shell is amorphous SiO_2_. C@SiO_2_ achieved its best performance when the mass ratio of TEOS and graphite used to prepare C@SiO_2_ was 1:1. This C@SiO_2_ has excellent thermal conductivity and good compatibility with cement, making it a thermally modified additive for TSCMs with outstanding performance.

C@SiO_2_ effectively ameliorates the deficiency of poor thermal conductivity in TSCMs. It enhances the thermal conductivity and improves the thermal storage efficiency of TSCMs, while also enhancing the thermal storage capacity per unit mass of TSCM. Moreover, higher doping levels of C@SiO_2_ correspond to better thermal performance of TSCMs. However, excessive C@SiO_2_ doping may adversely affect the fluidity and compressive strength of TSCMs, underscoring the importance of controlling the doping level.

The optimal doping level of C@SiO_2_ is determined to be 10%. Compared to a TSCM without C@SiO_2_, a TSCM with 10% C@SiO_2_ doping exhibits a 63.6% increase in thermal conductivity, an 11.2% increase in latent heat of phase change, and a 44.8% reduction in the time required to raise the temperature from 20 °C to 50 °C. Moreover, compared to ordinary cement mortar, the flow degree of this TSCM is reduced by 2.7%, while the 28-day compressive strength remained essentially the same, with no significant adverse effects on workability and mechanical properties. Consequently, it emerges as a promising cement-based energy storage material with commendable working performance, mechanical properties, thermal storage, and thermal conductivity.

## Figures and Tables

**Table 1:** Individual properties of MPCMs (%).

	Thermal Enthalpy	Initial Melting Temperature	Thermal Conductivity Coefficient
MPCM	124.6 J/g	22.8 °C	0.48 W/m·K

**Table 2:** Chemical composition of cement (%).

Oxide	SiO_2_	Al_2_O_3_	Fe_2_O_3_	CaO	MgO	K_2_O	Na_2_O	SO_3_	LOI
Cement	21.5	5.1	2.8	62.9	1.4	0.8	0.1	2.3	2.8

LOI: Loss on ignition.

**Table 3:** Raw material ratios for different C@SiO_2_ samples.

Code	Graphite (g)	TEOS (g)
S1	6	4
S2	5	5
S3	4	6
S4	3	7

**Table 4:** The mix proportion of TSCM samples.

Code	Cement (vol%)	MPCM (vol%)	C@SiO_2_ (vol%)	b/s	w/b
PC	100	0	0	1/3	0.5
0S	90	10	0		
5S	85	10	5		
10S	80	10	10		
15S	75	10	15		
20S	70	10	20		

(b/s) is binder-to-sand weight ratio; (w/b) is water-to-binder ratio.

**Table 5:** Elemental content of different samples (%).

No.	C	Si	O
S1	90.01	2.61	7.38
S2	81.78	4.88	13.34
S3	75.97	6.57	17.46
S4	65.88	9.16	24.96

## Data Availability

Data are contained within the article.
